# Arthroscopic lift, drill, fill and fix (LDFF) is an effective treatment option for primary talar osteochondral defects

**DOI:** 10.1007/s00167-019-05687-w

**Published:** 2019-09-13

**Authors:** Kaj T. A. Lambers, Jari Dahmen, Mikel L. Reilingh, Christiaan J. A. van Bergen, Sjoerd A. S. Stufkens, Gino M. M. J. Kerkhoffs

**Affiliations:** 1 Department of Orthopaedic Surgery, Amsterdam Movement Sciences, Amsterdam UMC, University of Amsterdam, Meibergdreef 9, 1105 AZ Amsterdam, The Netherlands; 2 Academic Center for Evidence Based Sports Medicine (ACES), Amsterdam, The Netherlands; 3 Amsterdam Collaboration for Health and Safety in Sports (ACHSS), AMC/VUmc IOC Research Center, Amsterdam, The Netherlands; 4 Department of Orthopedic Surgery, Amphia Hospital, Breda, The Netherlands; 5 Department of Orthopedic Surgery, Albert Schweitzer Hospital, Dordrecht, The Netherlands

**Keywords:** Ankle, Arthroscopy, Osteochondral defects, Fixation

## Abstract

**Purpose:**

The purpose of this study was to describe the mid-term clinical and radiological results of a novel arthroscopic fixation technique for primary osteochondral defects (OCD) of the talus, named the lift, drill, fill and fix (LDFF) technique.

**Methods:**

Twenty-seven ankles (25 patients) underwent an arthroscopic LDFF procedure for primary fixable talar OCDs. The mean follow-up was 27 months (SD 5). Pre- and post-operative clinical assessments were prospectively performed by measuring the Numeric Rating Scale (NRS) of pain in/at rest, walking and when running. Additionally, the Foot and Ankle Outcome Score (FAOS) and the Short Form-36 (SF-36) were used to assess clinical outcome. The patients were radiologically assessed by means of computed tomography (CT) scans pre-operatively and 1 year post-operatively.

**Results:**

The mean NRS during running significantly improved from 7.8 pre-operatively to 2.9 post-operatively (*p* = 0.006), the NRS during walking from 5.7 to 2.0 (*p* < 0.001) and the NRS in rest from 2.3 to 1.2 (*p* = 0.015). The median FAOS at final follow-up was 86 for pain, 63 for other symptoms, 95 for activities of daily living, 70 for sport and 53 for quality of life. A pre- and post-operative score comparison was available for 16 patients, and improved significantly in most subscores. The SF-36 physical component scale significantly improved from 42.9 to 50.1. Of the CT scans at 1 year after surgery, 81% showed a flush subchondral bone plate and 92% of OCDs showed union.

**Conclusion:**

Arthroscopic LDFF of a fixable primary talar OCD results in excellent improvement of clinical outcomes. The radiological follow-up confirms that fusion of the fragment is feasible in 92%. This technique could be regarded as the new gold standard for the orthopedic surgeon comfortable with arthroscopic procedures.

**Level of evidence:**

Prospective case series, therapeutic level IV.

## Introduction

Management of talar osteochondral defects (OCD) is still a challenge. Multiple factors are to be taken into account when determining the best available treatment. There are numerous surgical treatment strategies available, with a substantial increase in the number of procedures over the past decades. For primary talar OCDs, bone marrow stimulation is the most frequently performed treatment [5]. This technique has shown good clinical results at short-term and mid-term follow-up [5, 11, 17]. It aims at focusing on the intrinsic capacity of the ankle to heal the cartilage due to the formation of fibrocartilage [24]. However, previous studies have shown that the quality of fibrocartilage decreases over time, that it shows inferior wear characteristics, and it has been proven that the subchondral bone plate shows irregularities and depressions at follow-up; all of these factors potentially being the explanation why one-third of the patients show progression of ankle osteoarthritis at long-term follow-up [8, 20, 21, 26, 30, 34, 35].

Subsequently, a novel technique has been developed, the arthroscopic internal fixation technique of talar OCDs, having the theoretical advantage of preserving hyaline cartilage, restoring the original congruency of the ankle mortise, facilitating healing of the cartilage and the subchondral bone, and thus restoring subchondral bone plate quality, thereby potentially leading to reduced percentages of ankle osteoarthritis at follow-up [14, 28, 29]. Recently, the authors of the present study reported on a promising minimally invasive arthroscopic internal fixation technique, the lift, drill, fill and fix (LDFF) procedure. This is a novel technique used for arthroscopic fixation of primary and large (>10 mm) OCDs of the talar dome. Although the technique showed highly promising clinical and radiological short-term results, the study contained a limited number of patients [14].

Consequently, the aim of the present study is, therefore, to present the mid-term follow-up clinical and radiological results of the arthroscopic LDFF technique for large primary talar OCDs. The hypothesis is that the results of the short-term follow-up are similar in this extended patient cohort, are durable over time and show a high rate of fusion.

## Materials and methods

This study was approved by the local Medical Ethics Committee at the University of Amsterdam with reference number MEC 08/326, and it was performed in accordance with the current ethical standards (Declaration of Helsinki).

The indication for an LDFF technique was a large primary OCD of the talar dome with a diameter of more than 10 mm (anterior–posterior or medial–lateral) and a bony fragment of at least 3 mm in depth on computed tomography (CT). The patients had to have persistent deep ankle pain for more than 1 year. The contraindications were ankle osteoarthritis grade II or grade III [39], type IV (displaced) OCDs according to the modified Berndt and Harty classification scale [3, 19], rheumatoid arthritis, chondral lesions, a concomitant defect in the tibia, an ankle fracture < 6 months old, tendinopathy, advanced osteoporosis, concomitant painful or disabling disease of the lower limb and infectious pathology or any kind of malignancy.

### Pre-operative planning

Patients were assessed at the outpatient clinic pre-operatively. A pre-operative computed tomography (CT) scan was made to assure the diagnosis as well as to determine size, location, shape, morphology of the talar osteochondral defect and to determine arthroscopic (anterior/posterior) access of the defect.

### Operative technique

**Fig. 1:**
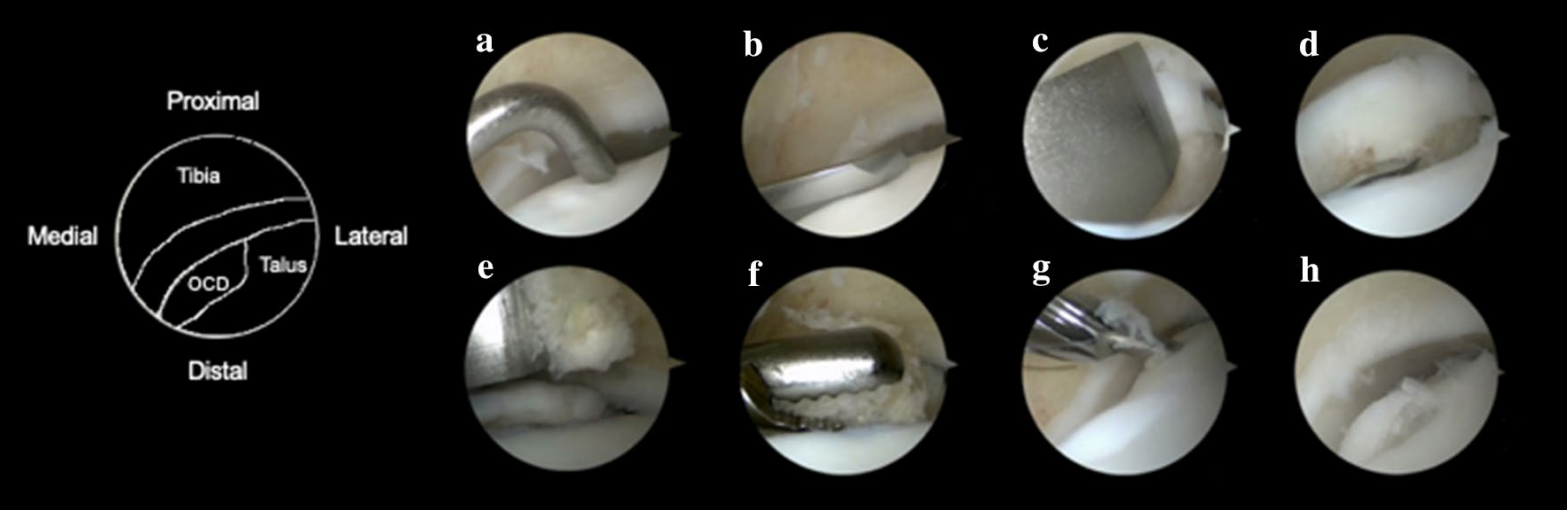
Arthroscopic LDFF technique. **a** A medial OCD is identified. **b** An osteochondral flap is created with use of a beaver knife. **c** With a chisel the osteochondral flap is lifted. **d** The bone flake is drilled with a K-wire to promote revascularization. **e** Cancellous bone is harvest from the distal tibia using a 4-mm chisel. **f** With an arthroscopic grasper the cancellous bone is transported into the defect. **g** A cannulated system allows predrilling and tapping of a compression screw (Arthrex Inc, Naples, USA). **h** an absorbable Bio-Compression screw is placed recessed relative to the surrounding surface of hyaline cartilage

All of the patients were operated by the senior author (GK) (Fig. 1). Procedures were carried out under spinal or general anaesthesia. Patients were placed in a supine position. Standard anteromedial and anterolateral portals were used. A standard 4-mm, 30° arthroscope was used, and arthroscopic portals were interchangeably used to achieve optimal vision of the defect. The location of the OCD was identified and then a beaver knife was used to make a sharp osteochondral flap. The posterior side of the flap was left intact and used as a lever. Consequently, this flap was lifted from anterior with use of a chisel (lift). Then the attached bone of the osteochondral flap and the osteosclerotic area of the bed were both debrided and drilled to stimulate revascularisation (drill). Any subchondral cysts were also curetted and drilled. With the use of a chisel, cancellous bone was harvested from the distal tibial metaphysis, and the defect was filled with these cancellous chips, transported into the defect with a grasper (fill). The last step consisted of fixating the fragment (fix) with either a Bio-Compression screw (Arthrex Inc., Naples, USA), with multiple chondral darts (Arthrex Inc., Naples, USA), or a combination of those two. Skin incisions were sutured with 3.0 Ethilon single stitches.

### Postoperative management

After surgery, all patients were placed in a short-leg cast and were kept non-weight bearing for 6 weeks post-operatively. Then the cast was exchanged for a short-leg walking cast. This cast was set in neutral flexion and neutral hindfoot position. At that time, full weight bearing was allowed. Flexion and extension exercises in between all cast changes were encouraged. After a total of 12 weeks of immobilisation, the cast was removed. Physical therapy was then prescribed to assist functional recovery.

### Patient cohort

The study group consisted of 25 patients, of whom 2 received an operation on both ankles, which resulted in a total of 27 operated ankles. The patients who received an operation on both ankles filled in a questionnaire for each ankle. The operation on the contralateral ankle was performed 1.5 year after the first surgery in one patient and after 1 year in the other patient. There were 14 males and 11 females. The median age at surgery was 17 years (range 11–63 years). The mean body mass index was 22. Eight patients were operated on their left ankle, 17 on their right ankle and 2 patients bilaterally. No previous surgery on any of the treated ankles was reported. One patient reported a bipolar chevron operation on the ipsilateral hallux due to a hallux valgus. Another patient reported a previous bone marrow stimulation treatment of an earlier osteochondral defect but on the contralateral ankle. The median follow-up was 27 months (range 18 to 43 months).

### Clinical analysis

Patients were assessed pre-operatively, and at 1 and 2 years post-operatively. At all moments, patients were requested to fill out a Numeric Rating Scale (NRS) for pain in rest, during walking and during running if possible. The NRS is an 11-point scale that represents the spectrum of no pain to the worst pain imaginable (0–10) [9]. Additionally, a numeral rating scale for satisfaction was used to capture personal satisfaction of the whole management procedure.

At the beginning of the inclusion period, the authors used a native language validated American Orthopedic Foot and Ankle Society (AOFAS) Score as a clinical outcome scale [15]. However, during the proceeding of this study, a systematic review on different reported outcome measures showed that a native language validated Foot and Ankle Outcome Score (FAOS) was the best available Dutch Patient Reported Outcome Measure (PROM) for assessing the clinical status of various foot and ankle injuries [40]. Therefore, we initiated the use of pre-operative and post-operative clinical evaluation by means of the Foot and Ankle Outcome Score (FAOS) [38]. Since this questionnaire also is a completely patient-based questionnaire and therefore can be taken without the interference of a clinician thereby giving it a highly logistic advantage, it was decided to use this as our ankle outcome scale for any further future documentation. Finally, the 36-item Short Form Health Survey (SF-36) was used to assess general quality of life. For all questionnaires, a version validated in the native language was used [32, 33, 38].

### Radiological analysis

Pre-operatively as well as at 1 year post-operatively, the patients were evaluated by means of a computed tomography scan (CT scan). Defects were classified according to the modified Berndt and Harty classification system [3, 19]. A nine-grid scheme as previously described by Elias et al. was used to assess the location in the talus for each osteochondral lesion [7]. Defect size was measured in three planes, including surface area and depth of the lesion. Post-operatively, the CT scans were scored on level of subchondral bone plate (flush/depressed) [8, 20, 21, 26, 30, 34, 35], and were scored on union rate of the fixated defect [14, 28, 29]. The scanning protocol involved ‘ultra high-resolution’ axial slices with an increment of 0.3 mm and a thickness of 0.6 mm. Multiplanar coronal and sagittal reconstructions were 1.0 mm.

### Statistical analysis

All statistics were performed with SPSS version 24 (IBM corp, Armonk, NY, USA). Categorical data are presented as frequency and continuous data as mean with SD or median with range, all depended on its distribution. The paired Student’s *t* test was used for comparison of the normal distribution of pre- and post-operative means. A Wilcoxon signed-rank test was used if data were skewed. The comparisons with *p* < 0.05 were considered to be statistically significant.

## Results

### Clinical outcome

In all patients, LDFF led to a significant improvement of the NRS of pain and the majority of the FAOS and SF-36 subscales. Of the 25 patients, 23 played sports before their injury. Of these, 20 were able to return to sports of which 7 played at a competitive level and 13 at a recreational level. The NRS significantly improved on each subscale (Table 1). Median satisfaction of the procedure was reported to be 8 (SD 3.3, range 0–10).

**Table 1:** NRS scores pre- and post-operative

	Pre-operative	Final follow-up	*p* value
NRS pain in rest	2.3 (SD1.8)	1.2 (SD1.8)	0.015
NRS pain during walking	5.7 (SD 2.6)	2.0 (SD2.5)	< 0.001
NRS pain during running	7.8 (SD1.8)	2.9 (SD2.6)	0.006

Results are expressed as mean ± standard deviation

**Fig. 2:**
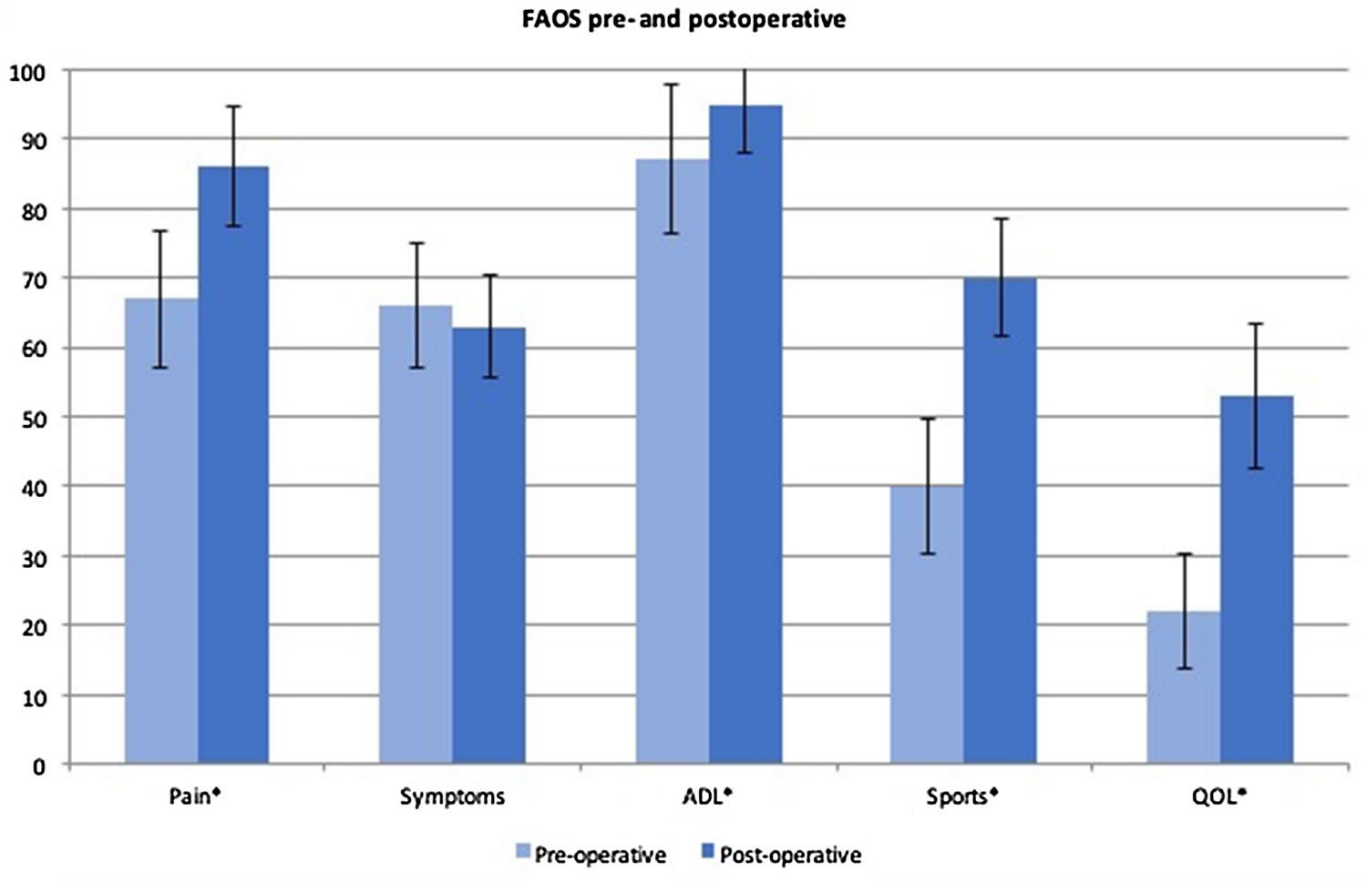
Pre- and post-operative FAOS Scores. *FAOS* Foot and Ankle Outcome Score, *ADL* activities of daily living, *QOL* quality of life; *significant *p* value

Although we were able to obtain all post-operative FAOS on all patients, the pre-operative FAOS was not available in 11 patients for reasons as stated above in the “Materials and methods” section, meaning that a pre-operative to post-operative comparison could only be performed in 16 cases. Pre- and post-operative scores for pain were 67 (SD 20) and 86 (SD 22), respectively (*p* = 0.026). For other symptoms, 66 (SD 18) and 63 (SD 19) (n.s.), for activities of living 87 (SD 22) and 95 (SD 18) (*p* = 0.029), for sport 40 (SD 20) and 70 (SD 22) (*p* = 0.002) and for quality of live, 22 (SD 17) and 53 (SD 27) (*p* = 0.006) all pre- and post-operative, respectively. FAOS are displayed in Fig. 2.

The Physical Component Scale of the SF-36 improved from a pre-operative score of 42.9 (SD 9.2) to 50.1 (SD 7.7) at final follow-up (*p* = 0.007). The Mental Component Scale decreased from 55.7 (SD 6.0) to 49.8 (SD 12.0), though not statistically significant.

### Radiological outcomes

All patients but one received a CT scan at their 1-year follow-up appointment. One patient did not show up at his post-operative CT scan appointment. The mean pre-operative OCD size was 14 mm (SD 2.8 mm) in the anterior–posterior direction, 8 mm (SD 2.2) in the medial–lateral direction and 7 mm (SD 3.1 mm) in depth. Twenty-three defects were located on the medial talar dome (13 at the center and 10 anterior) and 4 on the lateral talar dome (1 at the center and 3 anterior). Six defects were classified as partially fractured and thus stage II lesions. Twenty-one defects were classified as non-displaced fragments without attachment and, therefore, classified as stage III lesions.

**Fig. 3:**
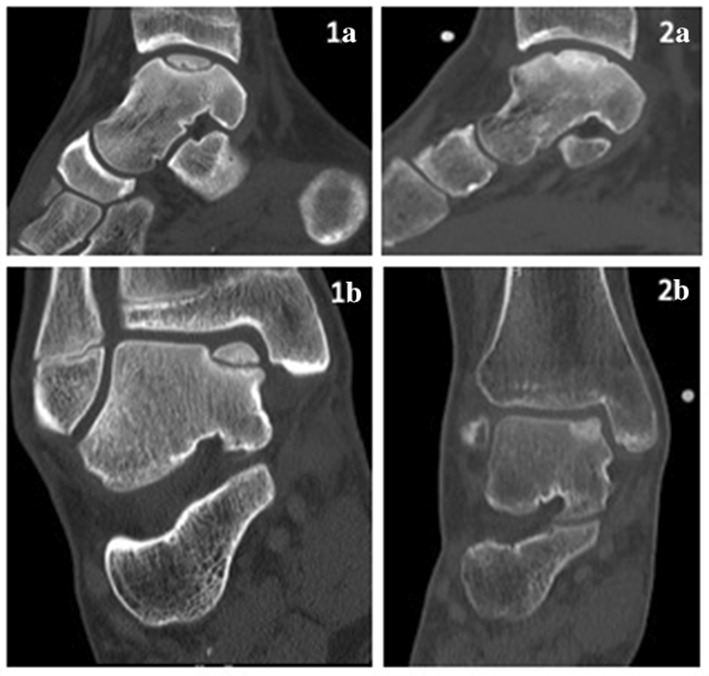
Example of a pre-operative sagittal (**1A**) and coronal (**1B**) CT scan of the OCD of a 15-year-old patient and sagittal (**2A**) and coronal (**2B**) CT scan from the same patient 1 year after the LDFF procedure

Of the 26 post-operative CT scans, 81% of the patients (21/26) showed a flush subchondral bone plate, and 92% (24/26) of OCDs showed union of the osteochondral fragment (Fig. 3).

### Complications

No serious adverse events occurred after the arthroscopic LDFF procedure, and there were no complications reported. One patient received a re-operation due to unsatisfactory results. This patient received a bone marrow stimulation operation 2 years after the initial LDFF surgery.

## Discussion

The most important finding of the present study was that we found satisfactorily outcome scores with a high fusion rate. Treatment of large OCDs by fixation has been shown to be a successful method both for acute and chronic lesions but the literature on this topic is scarce, and there are very few studies describing its clinical and radiological results [14, 16, 23, 27, 31]. This present prospective study describes the clinical and radiological outcomes of a previously published arthroscopic method of fixation, named the lift, drill, fill and fix (LDFF) technique [14]. It can be stated that, at a 2-year follow-up period, this technique shows a high rate of satisfaction, a significant decrease of experienced pain during weight-bearing activities such as walking and running, and it shows that a high rate of return to sports could be achieved. Furthermore, the present study showed that a significant improvement in the quality of life and physical well-being was observed.

Clinically, perceived pain decreased after surgery, especially during normal weight-bearing activities like walking. Although perceived pain during running also showed a significant drop post-operatively, with an average post-operative value of 2.9 there is still room for improvement, especially since most OCD patients are young and still very active, and thus there is high demand to return to their previous level of sport.

Numerous surgical options can be used to treat primary talar osteochondral defects. For the small defects, there is general consensus that a type of bone marrow stimulation (BMS) can be a successful treatment option yielding success rates around 82%, and high return-to-sports at short-term follow-up [5, 13]. In the literature, this is also the most described treatment option for primary osteochondral defects [5].

Arthroscopic BMS has been used for a long time and thanks its popularity to the relative simplicity, low costs and minimal invasiveness [1, 2, 4, 6, 10, 12, 22]. It aims at focusing on the intrinsic capacity of the ankle to heal the cartilage due to the formation of fibrocartilage [24]. Results are good in up to 80% of patients but the technique also gives concerns since this fibrocartilage shows decrease in quality over time with a possible increase in osteoarthritic changes [5, 6, 20]. In up to one-third (33–34%) of patients, a progression of ankle osteoarthritis can be seen at mid-term and long-term follow-up, and the subchondral bone plate seems depressed on CT analysis in about three-fourth (74%) after 1 year [8, 28, 35]. Also, second look arthroscopy showed an incomplete healing of the defect 1 year post-operatively in 40% of the treated patients [18].

From an evidence-based standpoint, it can therefore be concluded that BMS can be a successful treatment option in case of disappearance of the chondral layer of the talar dome, or in cases of severely damaged or ulcerated cartilage. However, in case of a fragmented defect of bone with intact overlying cartilage, a fixation technique to treat these defects can be highly suitable. The theoretical advantages of a fixation technique are the restoration of the subchondral bone and preservation of the hyaline cartilage. A recent publication by Reilingh et al. showed that the subchondral bone healing in patients operated by means of a fixation was significantly higher in comparison with patients being operated by means of a bone marrow stimulation procedure. The authors, therefore, share the opinion that a method of fixation is the preferred treatment of an osteochondral fragment with an intact overlying cartilage layer. For theoretical superior healing, it is necessary for the loose fragment to have an osseous part. Next to the biological advantages, the risk of an attempt of fixation is low since, if fixation would fail, it is still possible to perform a subsequent bone marrow stimulation procedure.

As surgical techniques develop and improve, the optimal way of fixation stays debatable. There is a growing number of fixation possibilities which consist of biocompression or steel screws and additional bioabsorbable darts or pins. Other described methods are K wires and bony pegs [14, 16, 23, 25, 29, 31]. Since numbers of patients are low in the included case series, it is difficult to make methodologically correct comparisons and, therefore, the optimal method of fixation should be evaluated for each individual case, taking into account all potential prognostic factors, the radiological pre-operative morphological characteristics of the defect and the individual wishes of the patient. An International Consensus Meeting on Cartilage Repair of the Ankle held in Pittsburgh in November 2017 showed that there was a strong consensus that if possible the fragment should be fixed with two devices with at least one being used for compression and the other to prevent rotation [29]. Fragments, however, may be very small and fragile, and double fixation will not always be possible without compromising the stability of the fragment.

The LDFF procedure as described above is an arthroscopic method and can be technically demanding. In our institution, with the gained experience over the past years performing this type of surgery, we also perform an open procedure—by means of a medial malleolar osteotomy—when the full potential of this procedure cannot be reached with an arthroscopic procedure alone. Location of the OCD and its arthroscopic accessibility has to be taken into account during pre-operative planning [36, 37]. We found that during arthroscopic treatment, it can often be difficult to find the optimal angle to fixate the fragment and create optimal stability. A recent study investigating the influence of the angle of pin insertion on the clinical outcomes concerning clinical efficacy of a fixation procedure for osteochondral defects of the talus showed that shallow pin insertion was significantly associated with osteolytic changes around the pins and persistence of bone marrow edema on MRI [23]. The patients with these radiological inferior outcomes, however, still had good to excellent results at 1 year of follow-up after the surgery. It is, therefore, highly important to weigh the pros and cons of the arthroscopic versus the open procedure in combination with a proper shared decision making with the patients, so that the proper surgical choice can be made.

This study should be interpreted in the light of its strengths and weaknesses. The study group is small but relatively large when compared to the previous study groups describing the results of fixation. Another disadvantage is some missing pre-operative FAOS. Since this questionnaire was validated after inclusion of the first patients, we started using this outcome measure during the study, resulting in the absence of 11 pre-operative FAOS. We changed to the FAOS whilst, at that moment, a systematic review about different reported outcome measures showed the FAOS as the best available Dutch PROM [40]. Further evaluation of the FAOS showed good responsiveness in patients 1 year after hindfoot and ankle surgery [33]. Since we were able to obtain all outcome measures at the 2-year follow-up, we believe this study shows representative results. The clinical relevance behind the work of the present study is that it raises awareness amongst orthopedic surgeons that fixating a fragment of an osteochondral defect in the ankle is an effective, safe surgical treatment option, and should be considered when treating these patients.

## Conclusion

Arthroscopic fixation by means of the Lift–Drill–Fill–Fix method for primary osteochondral defects of the talus results in excellent clinical improvement at 2-year follow-up and confirms radiological (CT) union in 92%. This novel technique could be regarded as the new gold standard for the orthopedic surgeon comfortable with arthroscopic procedures.

## References

[1] Alexander AH, Lichtman DM (1980). Surgical treatment of transchondral talar-dome fractures (osteochondritis dissecans). Long-term follow-up. J Bone Jt Surg Am.

[2] Bauer M, Jonsson K, Linden B (1987). Osteochondritis dissecans of the ankle. A 20-year follow-up study. J Bone Jt Surg Br.

[3] Berndt AL, Harty M (1959). Transchondral fractures (osteochondritis dissecans) of the talus. J Bone Jt Surg Am.

[4] Chuckpaiwong B, Berkson EM, Theodore GH (2008). Microfracture for osteochondral lesions of the ankle: outcome analysis and outcome predictors of 105 cases. Arthroscopy.

[5] Dahmen J, Lambers KTA, Reilingh ML, van Bergen CJA, Stufkens SAS, Kerkhoffs G (2018). No superior treatment for primary osteochondral defects of the talus. Knee Surg Sports Traumatol Arthrosc.

[6] Donnenwerth MP, Roukis TS (2012). Outcome of arthroscopic debridement and microfracture as the primary treatment for osteochondral lesions of the talar dome. Arthroscopy.

[7] Elias I, Zoga AC, Morrison WB, Besser MP, Schweitzer ME, Raikin SM (2007). Osteochondral lesions of the talus: localization and morphologic data from 424 patients using a novel anatomical grid scheme. Foot Ankle Int.

[8] Ferkel RD, Zanotti RM, Komenda GA, Sgaglione NA, Cheng MS, Applegate GR (2008). Arthroscopic treatment of chronic osteochondral lesions of the talus: long-term results. Am J Sports Med.

[9] Gagliese L, Weizblit N, Ellis W, Chan VW (2005). The measurement of postoperative pain: a comparison of intensity scales in younger and older surgical patients. Pain.

[10] Giannini S, Vannini F (2004). Operative treatment of osteochondral lesions of the talar dome: current concepts review. Foot Ankle Int.

[11] Hannon CP, Bayer S, Murawski CD, Canata GL, Clanton TO, Haverkamp D (2018). Debridement, curettage, and bone marrow stimulation: Proceedings of the international consensus meeting on cartilage repair of the ankle. Foot Ankle Int.

[12] Hannon CP, Smyth NA, Murawski CD, Savage-Elliott I, Deyer TW, Calder JD (2014). Osteochondral lesions of the talus: aspects of current management. Bone Jt J.

[13] Hurley ET, Shimozono Y, McGoldrick NP, Myerson CL, Yasui Y, Kennedy JG (2018). High reported rate of return to play following bone marrow stimulation for osteochondral lesions of the talus. Knee Surg Sports Traumatol Arthrosc.

[14] Kerkhoffs GM, Reilingh ML, Gerards RM, de Leeuw PA (2016). Lift, drill, fill and fix (LDFF): a new arthroscopic treatment for talar osteochondral defects. Knee Surg Sports Traumatol Arthrosc.

[15] Kitaoka HB, Alexander IJ, Adelaar RS, Nunley JA, Myerson MS, Sanders M (1994). Clinical rating systems for the ankle-hindfoot, midfoot, hallux, and lesser toes. Foot Ankle Int.

[16] Kumai T, Takakura Y, Kitada C, Tanaka Y, Hayashi K (2002). Fixation of osteochondral lesions of the talus using cortical bone pegs. J Bone Jt Surg Br.

[17] Lambers KTA, Dahmen J, Reilingh ML, van Bergen CJA, Stufkens SAS, Kerkhoffs G (2018). No superior surgical treatment for secondary osteochondral defects of the talus. Knee Surg Sports Traumatol Arthrosc.

[18] Lee KB, Bai LB, Yoon TR, Jung ST, Seon JK (2009). Second-look arthroscopic findings and clinical outcomes after microfracture for osteochondral lesions of the talus. Am J Sports Med.

[19] Loomer R, Fisher C, Lloyd-Smith R, Sisler J, Cooney T (1993). Osteochondral lesions of the talus. Am J Sports Med.

[20] Lynn AK, Brooks RA, Bonfield W, Rushton N (2004). Repair of defects in articular joints. Prospects for material-based solutions in tissue engineering. J Bone Jt Surg Br.

[21] Marsh JL, Buckwalter J, Gelberman R, Dirschl D, Olson S, Brown T (2002). Articular fractures: does an anatomic reduction really change the result?. J Bone Jt Surg Am.

[22] Murawski CD, Kennedy JG (2013). Operative treatment of osteochondral lesions of the talus. J Bone Jt Surg Am.

[23] Nakasa T, Ikuta Y, Tsuyuguchi Y, Ota Y, Kanemitsu M, Adachi N (2018). MRI tracking of the effect of bioabsorbable pins on bone marrow edema after fixation of the osteochondral fragment in the talus. Foot Ankle Int.

[24] O'Driscoll SW (1998). The healing and regeneration of articular cartilage. J Bone Jt Surg Am.

[25] Park CH, Choi CH (2018). A novel method using bone peg fixation for acute osteochondral fracture of the talus: a surgical technique. Arch Orthop Trauma Surg.

[26] Qiu YS, Shahgaldi BF, Revell WJ, Heatley FW (2003). Observations of subchondral plate advancement during osteochondral repair: a histomorphometric and mechanical study in the rabbit femoral condyle. Osteoarthr Cartil.

[27] Reilingh ML, Kerkhoffs GM, Telkamp CJ, Struijs PA, van Dijk CN (2014). Treatment of osteochondral defects of the talus in children. Knee Surg Sports Traumatol Arthrosc.

[28] Reilingh ML, Lambers KTA, Dahmen J, Opdam KTM, Kerkhoffs G (2017). The subchondral bone healing after fixation of an osteochondral talar defect is superior in comparison with microfracture. Knee Surg Sports Traumatol Arthrosc.

[29] Reilingh ML, Murawski CD, DiGiovanni CW, Dahmen J, Ferrao PNF, Lambers KTA (2018). Fixation techniques: Proceedings of the international consensus meeting on cartilage repair of the ankle. Foot Ankle Int.

[30] Reilingh ML, van Bergen CJ, Blankevoort L, Gerards RM, van Eekeren IC, Kerkhoffs GM (2016). Computed tomography analysis of osteochondral defects of the talus after arthroscopic debridement and microfracture. Knee Surg Sports Traumatol Arthrosc.

[31] Schuh A, Salminen S, Zeiler G, Schraml A (2004). Results of fixation of osteochondral lesions of the talus using K-wires. Zentralbl Chir.

[32] Sierevelt IN, Beimers L, van Bergen CJA, Haverkamp D, Terwee CB, Kerkhoffs G (2015). Validation of the Dutch language version of the Foot and Ankle Outcome Score. Knee Surg Sports Traumatol Arthrosc.

[33] Sierevelt IN, van Eekeren IC, Haverkamp D, Reilingh ML, Terwee CB, Kerkhoffs GM (2016). Evaluation of the Dutch version of the Foot and Ankle Outcome Score (FAOS): responsiveness and minimally important change. Knee Surg Sports Traumatol Arthrosc.

[34] Stufkens SA, Knupp M, Horisberger M, Lampert C, Hintermann B (2010). Cartilage lesions and the development of osteoarthritis after internal fixation of ankle fractures: a prospective study. J Bone Jt Surg Am.

[35] van Bergen CJ, Kox LS, Maas M, Sierevelt IN, Kerkhoffs GM, van Dijk CN (2013). Arthroscopic treatment of osteochondral defects of the talus: outcomes at eight to twenty years of follow-up. J Bone Jt Surg Am.

[36] van Bergen CJ, Tuijthof GJ, Blankevoort L, Maas M, Kerkhoffs GM, van Dijk CN (2012). Computed tomography of the ankle in full plantar flexion: a reliable method for preoperative planning of arthroscopic access to osteochondral defects of the talus. Arthroscopy.

[37] van Bergen CJ, Tuijthof GJ, Maas M, Sierevelt IN, van Dijk CN (2012). Arthroscopic accessibility of the talus quantified by computed tomography simulation. Am J Sports Med.

[38] van den Akker-Scheek I, Seldentuis A, Reininga IH, Stevens M (2013). Reliability and validity of the Dutch version of the Foot and Ankle Outcome Score (FAOS). BMC Musculoskelet Disord.

[39] van Dijk CN, van Bergen CJ (2008). Advancements in ankle arthroscopy. J Am Acad Orthop Surg.

[40] Weel H, Zwiers R, Sierevelt IN, Haverkamp D, van Dijk CN, Kerkhoffs GM (2015). Dutch-language patient-reported outcome measures for foot and ankle injuries; a systematic review. Ned Tijdschr Geneeskd.

